# Helping Mom Help Baby: Nutrition-Based Support for the Mother-Infant Dyad During Lactation

**DOI:** 10.3389/fnut.2020.00054

**Published:** 2020-04-21

**Authors:** Erin L. Ford, Mark A. Underwood, J. Bruce German

**Affiliations:** ^1^Department of Food Science and Technology, Foods for Health Institute, University of California, Davis, Davis, CA, United States; ^2^UC Davis Medical Center, Sacramento, CA, United States; ^3^Foods for Health Institute, University of California, Davis, Davis, CA, United States

**Keywords:** mother-infant dyad, precision nutrition, lactation, breastfeeding (BF), metabolism, premature Birth, microbiota (microorganism)

## Abstract

Lactation and breastfeeding support the short- and long-term health of both mother and infant, yet the success of these processes depend upon individual and combined factors of the pair. Complications during pregnancy and delivery greatly affect the likelihood that a mother will be capable of breastfeeding for at least the recommended 6 months. Guidelines for women regarding postpartum diet and lifestyle management also fail to reflect the diversity of mother-infant pairs and their circumstances. In our analysis of the literature, we have identified a categorical deficit in modern scientific discourse regarding human lactation; namely, that postpartum involves full-body contribution of resources and thus requires the application of nutrition from a systemic perspective.

**Graphical Abstract:**
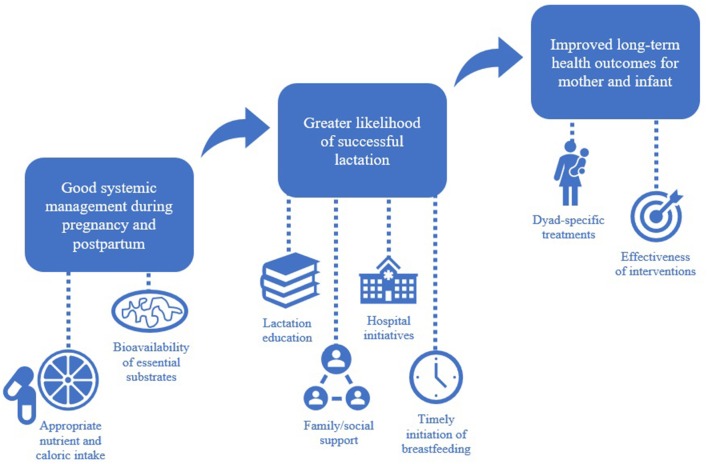
The contributing factors involved in achieving successful lactation are interdependent and unique to each mother-infant pair.

## Introduction

Breast milk provides a complex, dynamic, and targeted source of nourishment to an infant during the first months of life. Reductionist perspectives of lactation, however, tend to overlook the true diversity of interactions between a mother, her environment, and the growing infant. Each compositional change in breast milk reflects the influence of genetics, environment, health status of the mother, unique developmental needs of the child, and countless additional factors (1–4). Traditionally, lactation research in food science and nutrition has focused on milk's functionality as the exclusive food for infants. Milk composition varies extensively; it is distinct between women of different races (5, 6), between women of the same race over the course of lactation, and even within a single woman when considering diurnal effects and parity (7–9). Decades of research on the short- and long-term benefits associated with exclusive breastfeeding indicate a direct dose-dependent relationship between human milk consumption and reduced acute conditions in the infant including infection, diarrheal disease, and allergy; and chronic conditions including adult-onset diabetes, hypertension, autoimmunity, and obesity (10–15). A growing body of evidence also supports breastfeeding as a contributor to early structural brain development and consequent neurocognitive advantages (16, 17). Less is known about the benefits of lactation for the mother, although improved bone remineralization, more rapid return to pre-pregnancy weight, as well as decreased hypertension, hyperlipidemia, and cardiovascular disease was noted in a metadata analysis of postmenopausal women (18). Industrial development of artificial formula to be used in place of or in conjunction with human breast milk has bolstered a subfield of research primarily concerned with the elucidation of key compositional elements, and from outside milk's functional context of the mother-infant dyad. In reality, mother and infant operate synergistically. Breastfeeding and lactation remain largely undervalued, as evidenced by their scarcity in published academic literature and the lack of priority investment within public policy. From a public health perspective, only once the technologies and principles of scientific investigation address the integrative nourishment of the dyad as a whole can we begin to radically improve health outcomes and quality of life for future generations.

## Supporting Lactation Success

Modern precision nutrition has, until recently, been applied almost exclusively to the field of sports performance and the management of chronic diseases in adults. Advances in metabolomics technologies, which detect small metabolites in body fluids, have informed how the interaction between environment, microbiota, and host genetics and epigenetics contribute to the effect of an individual's diet on energy utilization and disease status (19). Still, there exists an enormous need for an approach to nutrition science that fundamentally emphasizes the achievement of calculable outcomes for a specific stage of life, physiological event (e.g., postpartum and lactation), or environment. Lactation, defined as the collection of innate functions necessary for the provision of adequate milk and coordination between maternal and infant systems, constitutes a key measure of success for the mother-infant dyad and an optimum target for novel precision medicine strategies. Employing one or more nutritional, social, or therapeutic interventions may be necessary in order to support lactation success.

Discourse in the fields of food science and nutrition tends to adopt a reductionist perspective aptly branded “nutritionism” by contemporaries in the field, wherein food is considered merely in terms of the sum of its macro- and micronutrient composition. Scholars agree that commercialization of nutrition principles, together with consumer exploitation by the health food industry and popular media, have obfuscated the relationship between diet and health (20, 21). There remains a lack of targeted, scientifically accredited guidelines for achieving long-term wellness in the midst of the modern chronic disease epidemic despite an abundance of well-intended claims. By extension, new mothers receive little informed advice regarding how to best nourish themselves and their baby during breastfeeding and beyond.

At present there exist no standardized means of assessing the natural progression of human lactation or defining optimum milk composition for a given infant; thus, a critical scientific objective centers on the establishment of metrics and reference values for specific infant populations (22). Clinical consideration for the broad range of maternal and neonatal requirements during the postpartum period and lactation by means of routine comprehensive evaluation—including routine analysis of the health of the dyad, emotional health of the mother, growth of the infant, and assessment of the microbiota and metabolome of the mother-infant pair—demonstrates the greatest potential for long-term health benefits for the dyad.

## The Postpartum Mother-Infant DYAD

The mammary, from a biological and physiological perspective, functions as a specialized bioreactor. Lactation involves all tissues and myriad biological processes of the mother. Under ideal circumstances, lactation also includes cooperation with and feedback from the infant. Meeting the demand of lactation depends upon a critical homeostatic maintenance of energy at the cellular level, a feat that involves efficient recruitment and utilization of essential compounds for communication between mother and infant. It follows that any internal or external perturbation can influence lactation and, consequently, the net success of the mother-infant dyad. Here we propose a more comprehensive view of lactation and breastfeeding which incorporates the dyad as a whole through the first years of life.

### The Microbiome of the Mother-Infant Dyad

Long-term programming begins *in utero* with maternal dietary and environmental exposures influencing the fetal immune system and continues with early communication between a mother, her milk, and the shared microbiota (23). During the first hours to days of life, a baby's gut rapidly acquires ambient bacteria, and it is during this time that dominance is established (24). The recent global focus on health consequences of early microbial colonization has facilitated the realization of several emergent themes. Theme 1: early exposure to *Bifidobacterium longum* subspecies *infantis* in combination with human milk feeding leads to swift colonization and domination of that specific strain within an infant. So long as breastfeeding remains the primary feeding regime, *B. infantis* persists as the keystone bacteria. Theme 2: a dramatic change in composition of the fecal microbiome of the breast-fed infant over the last century is characterized by an increase in fecal pH is associated with decreases in *Bifidobacterium* species and increases in *Enterobacteriaceae* and *Clostrideaceae* (25). In high-resource countries, half of the bacteria colonizing the infant gut are from non-maternal sources in the first four months of life (26). Theme 3: the absence of a single colonizer facilitates an erratic progression of microbes. In spite of exclusive breastfeeding this ecological community never achieves stability.

Sterile birth—which contrasts starkly with the microbially dense environments of human births prior to the twentieth century—has not yet been fully evaluated in terms of its evolutionary consequence. Exposure of the neonate to candidate bacterial colonizers clearly directs the trajectory of future microbial composition. In this way, microbiota perturbations that result from initial exposures to the extra-uterine environment can incur lasting effects. Whereas, *B. infantis* and other *Bifidobacteria* subspecies historically inhabited the gut of breastfed infants, the former now exists in a remarkably low fraction of babies in developed nations (27, 28). A recent comparison of two related subspecies showed more *B. infantis* in samples from infants in rural Indonesia and more *B. longum* subspecies *longum* in samples from infants in urban New Zealand, and that these differences are heavily related to breastfeeding practices (29). *Bifidobacterium infantis* (unlike *B. longum*) has been shown to preferentially consume human milk oligosaccharides and, when provided with this medium, will prevail when inoculated in competition with other bacteria (30). This critical contribution to the immunological integrity of infants supports *B. infantis* as distinctly “milk-oriented.” Further, administration of *B. infantis* to healthy breast-fed term infants eradicates differences in the fecal microbiota due to birth mode while also reducing levels of fecal Enterobacteriaceae that contain bacterial virulence factors and carry antibiotic resistance genes, resulting in decreased markers of intestinal inflammation (31–33).

Breast milk also facilitates vertical transfer of bacteria from the maternal gut to the neonatal gut, while potentially aiding in the establishment of genus-level dominance (34). Comparison of mother and infant fecal bacterial communities over time reveals a diversity of species and conspecific strains peaking in the first hours following birth and declining in diversity and polymorphic characteristic soon thereafter. Notably, vertical transfer of conspecific strains appears to be the most evolutionarily auspicious form of colonization, as evidenced by the superior retention of maternally derived gut microbes compared to foreign strains (26).

### Transfer of Innate and Adaptive Immunity

Immature immune function and abundant bacterial exposure characterize early infancy. Transfer of maternal immunity to her neonate begins *in utero* with immunoglobulin G through the placenta and maternal antimicrobial proteins and peptides through swallowed amniotic fluid. Short-chain fatty acids produced by gut bacteria contribute to adaptive immunity and the assembly of myriad protective factors that can be exchanged between maternal and infant systems (35). Immunization of the mother is also a safe and effective means of protecting the neonate early in life. Administration rates for influenza and pertussis—two vaccines currently recommended during pregnancy—remain low (36). Given this observation in conjunction with the development of several novel vaccines, evidence strongly suggests that vaccination offers a promising intervention for reducing infant mortality (37). Composition of the microbiota and its influence on vaccine response in infants and children demonstrates that breastfeeding continues to assist in immune maturation beyond the neonatal period (38). Transfer of immunity continues after birth with milk components including immunoglobulins, lactoferrin, and lysozyme; thus, breast milk facilitates mother-infant interactions that signal higher order metabolic, neurological, developmental, and immunological processes in the infant (39).

## Factors That Compromise Postpartum Mother-Infant Interaction

Designing strategies for a mother and her baby during the critical period immediately following parturition requires consideration of key circumstances, including both the ideal and the conspicuously non-ideal. Since breast milk provides a direct vehicle for transmission of nutrients and bioactive molecules, it is appropriate to first evaluate innate obstacles in its production and provision.

### Environmental and Physiological Barriers to Breastfeeding

Of the 80% of mothers who initiate breastfeeding, fewer than 25% maintain through the first 6 months postpartum, as is recommended by the American Academy of Pediatrics and the World Health Organization (40, 41). Failures in meeting these public health recommendations are often multicausal and structurally rooted. For instance, it has been shown that lack of evidence-based educational programs and family support are the top determining factors that govern whether a mother continues to breastfeed beyond hospital discharge (42). Despite a tremendous shift in family dynamics and workforce composition since the beginning of the twenty-first century, there currently exists no federally mandated minimum for employer-paid maternity or paternity leave in the United States. Many hospitals also routinely provide formula supplements to mothers who have not explicitly expressed a preference to breastfeed, which greatly increases the likelihood of more frequent formula feeding and the corresponding reduction in health benefits associated with exclusive breastfeeding (43). Socioeconomic confounders continue to influence the quality and level of care to which a mother has access, while disproportionate allocation of resources places hardship on financially disadvantaged families.

The decrease in rates of childbirth for women in their teens and twenties occurring coincidentally with an increase in births to women in their thirties and forties has raised the national average for maternal age. As of 2016, the mean age of a mother at the time of the birth of her first child achieved an all-time high of 26.6 years (44). These nationwide trends have introduced a new consideration within the field of lactation and postpartum care; namely, maternal age may influence metabolism and energy investment involved in childbearing and breastfeeding. Parity, which often but not exclusively indicates a history of breastfeeding, also contributes to lactation outcomes. Primiparous mothers generally experience greater difficulty in initiating and maintaining milk supply compared to multiparous mothers during the same period of time and who express a similar intent to breast feed (45). Breastfeeding success is subject to the influence of maternal health status as well. A medical history of mammary damage (augmentative or reductive surgeries, cancer, etc.), birth trauma, and coincident morbidities including diabetes and obesity affect the ability of a mother to produce and express milk for her baby. Reduction in milk volume and rate of synthesis may be temporary or persist throughout lactation (46–52).

Under any physiologically realistic setting, lactation never operates at 100% efficiency. In this sense, the mother-infant dyad is an inherently imperfect system. The discrepancy between the volume of milk that is synthesized and that which is removed is a unique characteristic of each mother-infant pair, subject to feedback mechanisms that regulate the mammary based on breast fullness as well as physical and hormonal stimulation by the nursing infant (22).

In some cases, the use of pharmaceutical or recreational drugs may contraindicate breastfeeding because of known or potential risk of transfer from the maternal circulation into her milk supply. Generally, compounds of low molecular weight and low protein binding ability; those that remain in high levels in maternal plasma; and those that readily cross the blood brain barrier are prone to mobilization into breast milk (53). Additionally, drugs that alter hormonal regulation of lactation contribute to complications at all stages of breast milk production. Resources such as the Drugs and Lactation database (LactMed®) from the National Library of Medicine and *Hale's Medications & Mother's Milk 2019* (53) provide a comprehensive examination of common substances and the known effects on the mother and infant associated with use during breastfeeding. The diversity of risks documented in the literature pertaining to breast milk contaminants justify support for more rapid, accurate, and sensitive methods for assessing milk composition in a clinical setting. Such analytics would inform conversation between a mother and her care team to advise whether breast milk alternatives might be the appropriate option when cessation of drug use is not possible or desirable.

### Preterm Birth and Congenital Abnormalities That Preclude Breastfeeding

Early mother-infant contact is limited when a neonate requires immediate medical attention, as is often the case when infants are born preterm or with congenital anomalies such as diaphragmatic hernia, gastroschisis, intestinal atresia, hypoxic-ischemic encephalopathy, severe forms of cyanotic congenital heart disease, Pierre Robin sequence, and other genetic syndromes. Evidence in support of the benefits of skin-to-skin holding—including dose-dependent improvement in the success of initiating first feed and duration of exclusive breastfeeding—have contributed to increases in rates of breastfeeding at discharge in many hospitals (54–56). Still, the acts of pumping, tube-feeding and feeding at the breast for many infants in the NICU remains arduous. Onset of lactation tends to occur much later in mothers of preterm infants, with colostrum not becoming available until as late as three days postpartum (57, 58). Even when lactogenesis II coincides with parturition (and the subsequent ejection of the placenta), failure to express milk within the first several hours following delivery hours may limit subsequent production (59). Together, these obstacles hinder breastfeeding success and, ultimately, compromise positive health outcomes for the mother-infant dyad.

### Maternal Obesity and Breastfeeding

Multiple studies have demonstrated an association between pre-pregnancy overweight and reduced initiation and maintenance of breastfeeding (60, 61). Contributing factors include increased incidence of prolonged labor and cesarean delivery, both of which delay lactogenesis. Ultimately, obese mothers are simultaneously the most likely to benefit from breastfeeding and the least likely to exclusive breastfeed. Research and interventions aimed at reversing this trend remain woefully absent.

## Nourishing the Mother-Infant DYAD

The distinct needs of a woman during pregnancy and lactation have prompted domestic and international regulatory agencies to endorse dietary guidelines for mothers based on prevailing nutritional consensus. These recommendations include warnings for substances that are known to cause harm to a developing fetus or breastfed infant and should therefore be avoided by women until cessation of breastfeeding; however, women differ considerably in how they implement dietary changes. Evidence-based advice often competes with popular opinion or culturally rooted practices, which in turn perpetuates misinformation and exacerbates confusion regarding health and nutrition. In creating a more comprehensive approach to nourishment of the mother-infant dyad it is important to consider the resources to which a mother might currently have access.

### Current Dietary Recommendations

Diet management for lactating women largely centers on the increased demand for calories and essential nutrients required to sustain production of a sufficient volume of breast milk that is also of high-quality composition. Increasingly, the consequences of unbalanced nutrition to the health of the mother-infant dyad must be evaluated alongside those of undernutrition. A mother expends an average of 500 kcal per day breastfeeding, with greater expenditure during later stages of lactation (62–64). This deficit amounts to a weight loss of one pound per week if the effects of additional dieting or exercise are omitted. On a purely energetic basis, expending 500 kcal per day should rapidly return a mother to pre-pregnancy weight, though recent evidence suggests that such normalization of body weight and composition is not universal (65). Mobilization of triglycerides alone at this magnitude undoubtedly requires systemic management of metabolic processes. Failure to recruit maternal energy stores for breast milk indicates that additional factors influence whole body fuel metabolism during lactation. Imbalances in these key metabolic signals may contribute to the risk of maternal obesity and explain the observation that mothers who breastfeed lose body fat more rapidly during the postpartum period compared to those who supplement breast milk with formula or other foods. The increase in widespread occurrence of metabolic dysregulation in the general population persists as one of the foremost health-related catastrophes of the twenty-first century. Lactating mothers likely experience the very same issues, with destructive effects on the progress of lactation and the quality of their milk. Indeed, altering the trajectory of global health for future generations means including lactation recovery as an essential element of postpartum care.

Precision nutrition strategies for lactation have yet to be implemented in public health policy despite their commercial availability elsewhere. Action in this direction would necessitate an understanding of the diversity among lactating women and their nutritional responses as opposed to the circumstances of lactation alone. The United States Department of Agriculture (USDA) currently recommends a modified “MyPlate” approach to maternal nutrition, with the acknowledgment that lactation requires increased intake of certain micronutrients, via multivitamin or diet, in order to reduce the risk of maternal and infant deficiencies (63). The most recent specifications focus on greens and starchy vegetables; fruits with high water and potassium content; fortified cereal grains; low-fat and calcium-rich dairy products; and a variety of plant- or animal-based protein sources while minimizing intake of seafood that tends to contain toxic levels of mercury. Additionally, the USDA advises that women limit added salt and select options that are high in iron, antioxidants, and omega-3 fats. Caffeinated, alcoholic, and sugar-sweetened beverages are suggested to be consumed infrequently (66). In 2018 the Center for Disease Control published updated guidelines emphasizing many of the same principles, referencing the USDA's “MyPlate” as an appropriate tool for evaluating individual requirements (62). The Children's Hospital of Philadelphia (CHOP), a leading center in breastfeeding and neonatal care, developed a more generalized plan for mothers to consult when making dietary selections while breastfeeding. This plan emphasizes the importance of variety while assessing how certain foods consumed by the mother may affect the infant on a case-by-case basis (67).

In 2001, the World Health Organization (WHO) published a booklet aimed at educating women about diet during pregnancy and lactation. These recommendations rely heavily on high-fiber carbohydrates as a main source of calories, as was recommended by the USDA until the twenty-first century revisions that shifted focus to fresh fruits and vegetables. Even then, plant-based foods are recommended above dairy and meat. Animal products are suggested as a supplementary means of acquiring vitamins and minerals that cannot be obtained from plant foods. According to this bulletin, low-fat options are preferred as a means of reducing cardiovascular risk, a precaution rooted in the global approach to eradicating heart disease in a primarily adult male population (68). Furthermore, diagnostics for determining cholesterol and assessing cardiac risk have been ubiquitously available for decades while no such measures have been attempted for routinely analyzing the composition of mother's own milk or the quality of diet as it pertains to lactation outcomes. Given the nature of public dietary targets it is clear that government agencies have not adequately prioritized lactation as warranting unique nutritional guidance. No updated editions from WHO have been made available to the international public.

Despite the global shift away from meat, dairy, and grains as predominant diet components, nutritionists and other healthcare professionals remain largely undecided regarding the adequacy of vegetarian or vegan diets for the health of both mother and infant during pregnancy and postpartum. Indeed, poorly planned plant-based diets restrict or eliminate sources of essential vitamins and minerals even while consuming plentiful calories. For lactating women who wish to continue with a lifestyle that omits some or all animal products, carefully monitoring food intake and fortifying with supplements is essential to the success of lactation and overall well-ness for the dyad.

#### Belief-Based Food Restrictions During Lactation

Purposeful omission of certain foods during breastfeeding is a common practice and is often motivated by historical customs, anecdotal claims, or popular media. It is important to distinguish verified dietary restrictions, allergies, or aversions which may or may not have been present before pregnancy from those that are voluntarily imposed. Sensitivities and adverse reactions in a mother or her baby in response to a particular food should always be monitored carefully by a care provider, but avoidance of entire classes of food based on scientifically unfounded or outdated claims may result in suboptimal nutrition, which can negatively affect the mother-infant dyad.

In a 2012 survey of lactation consultants based in the United States, researchers found that ~44% were familiar with folklore related to contraindicative foods perpetuated throughout the field, though only a minor percentage passed on this advice to patients (69). Several commonly avoided foods are those believed to promote allergy, colic, and gas in infants. Development of atopy in children has long been associated with the introduction of allergens from the maternal diet into breast milk. Whether or not these allergens result in morbidity has been the subject of controversy. Early introduction to peanuts from the maternal diet while breastfeeding has demonstrated both protective and adverse effects on the development of allergy in infants (70). Okan et al. (70) reported that the mothers of infants with colic tended to consume fewer grapes and lemons compared to the mothers of infants without colic. Furthermore, duration of crying episodes in the infants with colic was positively correlated with total dietary protein and negatively correlated with banana consumption, though both were of weak association and these findings have not been corroborated in the literature. One study noted positive associations between maternal consumption of cruciferous vegetable, bovine milk, onion, and chocolate and colic symptoms in breastfed infants (71). Yet another concluded that avoidance of bovine milk and eggs showed no effect on infant outcomes with regard to allergy prevention or risk (72). Alcohol has historically appeared on both sides of the argument in literature and popular media, with some alcoholic beverages even being marketed toward lactating women as a means of augmenting low milk supply (73). Alternative findings have indicated that moderate alcohol consumption during lactation temporarily inhibits oxytocin production and the consequent reflex ejection of milk from the mammary despite a corresponding increase in prolactin. Together, these physiological effects of endocrine disruption can compromise milk supply (74).

Prelacteal feeding—or the provision of a substance other than mother's milk to a neonate prior to the first breastfeeding—remains a common practice among Islamic and Hindu populations worldwide. In some instances, women may forego colostrum altogether and instead provide nourishment via water, sugar solutions, or herbal mixtures. Particularly within small communities, beliefs about colostrum being unclean or insufficient in volume have persisted (75). Religious convention reinforces long-held beliefs surrounding breast milk and infant nutrition, but in some cases prelacteal feeding and the ideologies regarding first milk can be harmful. Infants who don't receive the immune-fortifying compounds found in colostrum are at an increased risk of acute infection and related morbidities. Women who engage in prelacteal feeding also tend to initiate breastfeeding later. A hospital-based study conducted in India established a significant association between a delay in initial breastfeed and prelacteal feeding practices. Provision of mother's milk within the first hour postpartum confers the greatest benefit to the newborn, and prelacteal feeding can present an obstruction. Maternal education, birth mode, gestational age, antenatal breastfeeding counseling, and prelacteal feeding were determined to be the most influential factors associated with predicting timely initiation of breastfeeding (76). It is important to consider the sanctity of ceremonial traditions surrounding childbirth in many religions. By providing maternal education and support during the early postpartum period it is possible to advocate for the welfare of a mother and her infant while at the same time respecting the wishes of the family.

#### Toxicological Hazards During Lactation

As high-level consumers in the food chain, humans risk ingesting environmental substances that may harm an infant through breast milk, not unlike contraindicative drugs discussed previously. Predatory fish are known to bioaccumulate methyl mercury and other persistent organic pollutants, with concentration being directly related to life span, size, adiposity, and predation status of the fish (77). Generally-speaking, even low-mercury containing fish are advised to be consumed no more frequently than three times per week. Seafood with the highest tested mercury content such as king mackerel, orange roughy, swordfish, and tilefish are recommended to be avoided entirely by women while breastfeeding (78). Farmed fish and the increasingly prevalent practice of fishmeal feeding appears to exacerbate the aggregation of environmental toxins and increase human exposure (77). Organic mercury is both highly absorbed from maternal diet but also readily transferred into breast milk where it can disrupt cognitive development in infants (53).

### Milk Production as an Indicator of Lactation Success

The ultimate objective of lactation is to foster development of the infant while simultaneously supporting longevity and wellness of the mother. Breastfeeding encourages success of the mother-infant dyad, but only if all adaptations facilitate mutual benefit. In many cases it is important to consider ancillary measures when the demands of breastfeeding exceed the capacity of a mother to effectively provide for her infant.

#### Clinical Use of Galactagogues

Actual or perceived insufficient milk supply is one of the most common reason for terminating exclusive breastfeeding prior to 6 months postpartum (79, 80). Contrary to many women's assumptions, neonates require a small volume of milk in the first days of life. Considering that an infant's stomach capacity is <20 mL at birth, he or she initially requires mere teaspoons per feeding session (81). Still, in the event of delayed onset of lactation or prolonged low milk supply, galactagogues are commonly used in a clinical setting to stimulate milk production. Domperidone and metoclopramide, both of which primarily act to facilitate gastric emptying and relief of indigestion, are alternatively prescribed as pharmaceutical galactagogues due to their ability as dopamine antagonists to acutely increase prolactin levels (82, 83). Domperidone is currently not approved for use in the United States due to an increased risk of cardiovascular death associated with long-term use, while metoclopramide now carries a black box warning to emphasize the risk of tardive dyskinesia (84). Additionally, both have been noted to negatively impact milk production after cessation of use (53, 82).

Botanical galactagogues (classified as such to distinguish them from pharmaceutical counterparts) exist as popular alternatives to traditional medicine, persisting throughout Western medical practice and among support organizations despite unsubstantiated clinical evidence of efficacy. These substances are considered dietary supplements and are therefore not regulated for purity and potency by the FDA. Fenugreek is widely available and abundantly utilized globally as a botanical galactagogue. It does not appear to present any risk of acute toxicity (53, 85), though its physiological mode of action remains undetermined and the effects of long-term usage have not been evaluated. To date, only anecdotal reports and non-placebo-controlled studies have been published in the literature regarding fenugreek's touted galactagogic capacity. Recommended dosages provided by fenugreek manufacturers typically exceed 6,000 milligrams per day, and users often note a characteristic maple syrup scent in sweat and urine. Several case reports have been published describing extensive analysis of infants for maple syrup urine disease ultimately related to maternal fenugreek intake, underscoring the need for a careful history of maternal intake (86, 87). Risks associated with peanut allergy have been noted (53). In a small (*n* = 26) convenience-sampled study conducted on the effects of fenugreek supplementation on prolactin levels and milk production in mothers of preterm infants, a dosage of 1,725 milligrams daily failed to yield statistically significant changes to either metric (88).

Several peptidic compounds that act as oxytocin receptor agonists have recently demonstrated the potential for use in stimulating milk production, particularly for mothers of premature infants. A recent clinical trial (ongoing at the time of publication) is evaluating the effectiveness of a drug marketed as Merotocin, which selectively binds to a single oxytocin receptor, reducing the likelihood of acute hyponatremia associated with excess use and preventing collateral endocrine perturbation (89).

#### Supporting Postpartum Metabolic Demand

Current nutrition strategies applied to lactation primarily center on dietary intervention as a means of increasing the availability of essential nutrients required for milk production in addition to pharmacological support for hormonal stimulation of the mammary. The mammary operates inextricably from the rest of the body; therefore, metabolic insufficiency is expected to directly interfere with lactation success even with an adequate diet and complete biological development. For a variety of reasons, a mother may be metabolically incapable of assembling and transporting the substrates necessary for robust production. An alternative and complementary strategy involves regulation of whole-body metabolism and nourishment of diverse processes for systemic support of lactation.

Physiological, immunological, or metabolic stresses as well as birth method, infectious state, and maternal age all compromise a mother's metabolic performance. The most compelling evidence in support of targeted metabolic interventions for influencing the trajectory of lactation and outcomes of the mother-infant dyad is that of the response to nicotinamide riboside in lactating mice (90). Parturition and lactation demand considerable recruitment from maternal energy stores. Depletion of these stores, which exist in the form of NADH and its variants, reduces biomolecule synthesis and mobilization required for routine assembly of milk components. Production and utilization of energy depends upon the efficiency and availability of energy-carrying molecules. In a recent publication by Ear et al., postpartum metabolic stress was modeled in rat dams. Supplementation with nicotinamide riboside, a bioavailable NAD^+^ precursor, improved maternal response to postpartum via maintenance of lean body mass with a corresponding net improvement in volume and qualitative measures of milk consistent with improved maternal nutrient mobilization. Remarkably, the accompanying evidence supports observations of developmental, behavioral, and neurocognitive benefits in the pups that lasted into adulthood (90).

### Non-dietary Interventions to Improve Breastfeeding Success

Limited research supports meditation, yoga, exercise, and improved sleep quality as potential strategies for prevention and treatment of maternal stress, anxiety, and depression (91–97). Available studies suggest that regular moderate exercise during lactation improves cardiovascular fitness, plasma lipids, and insulin response. Further, exercise as an intervention during the lactation period does not negatively impact milk composition or volume, or maternal bone density (98–100). With the exception of a single proposed trial (101), we were unable to find any conclusive studies investigating the impact of the aforementioned strategies on breastfeeding success.

## Conclusion

Lactation-specific conditioning provides a unique opportunity to fundamentally alter long-term health trajectories for both mother and infant. Alleviating chronic disease and morbidity in adulthood requires prioritizing health at the beginning of life, and yet prevailing standards for nutrition remain problematically oversimplified to the point of being largely inapplicable.

Modern research efforts in human health and nutrition have placed near-exclusive emphasis on the benefits of specific foods or their constituent compounds as a panacea for acute and chronic disease. Health claims regarding the benefits of “superfoods” and nutraceuticals often center upon the notion that exogenous nutrients are taken up and seamlessly incorporated to impart a predictable, desired effect. In reality, biological systems are not universally equipped to utilize all currency equally (102). Current research continues to address cellular substrates and co-factors as targets for nutritional intervention strategies, as these directly influence the production and utilization of energy at a fundamental level. If performance output is assessed as a discrete and integrative indicator of total body nutrition, this focal shift would lead a new monumental wave of precision medicine. Ultimately, increasing efficiency would reduce disease and metabolic stress indicators while improving overall success during lactation and beyond.

Optimizing lactation for each mother-infant dyad requires a paradigm shift to dramatically expand research, education, and resources with this goal in mind. At-risk dyads, such as those affected by obesity, diabetes, preterm birth, and congenital anomalies, warrant exceptional dedication to care and demonstrate the greatest capacity for dramatic improvement. It is increasingly essential that prospective research efforts contribute to the development of new methods and technologies for evaluating nutritional and metabolic status. Furthermore, these advancements should coincide with the adoption of new policy to replace outdated practices and bolster timely accessibility to mother-infant pairs.

## Author Contributions

EF prepared, organized, and edited the manuscript from its initial state, and created the figure. MU provided critical feedback and contributed entire sections specific to his field of knowledge and medical practice. All authors revised and approved of the final manuscript. JG provided directive commentary and assisted in editing manuscript drafts.

## Conflict of Interest

The authors declare that the research was conducted in the absence of any commercial or financial relationships that could be construed as a potential conflict of interest.
